# Dietary Intake, Body Composition, and Oral Health Parameters among Female Patients with Primary Sjögren’s Syndrome

**DOI:** 10.3390/nu10070866

**Published:** 2018-07-04

**Authors:** Marianne B. Nesvold, Janicke L. Jensen, Lene H. Hove, Preet B. Singh, Alix Young, Øyvind Palm, Lene Frost Andersen, Monica H. Carlsen, Per Ole Iversen

**Affiliations:** 1 Department of Nutrition, Institute of Basic Medical Sciences, University of Oslo, 0317 Oslo, Norway; m.b.nesvold@studmed.uio.no (M.B.N.); l.f.andersen@medisin.uio.no (L.F.A.); m.h.carlsen@medisin.uio.no (M.H.C.); 2 Department of Oral Surgery and Oral Medicine, Faculty of Dentistry, University of Oslo, 0317 Oslo, Norway; j.c.l.jensen@odont.uio.no (J.L.J.); p.b.singh@odont.uio.no (P.B.S.); 3 Department of Cariology and Gerodontology, Faculty of Dentistry, University of Oslo, 0317 Oslo, Norway; l.h.hove@odont.uio.no (L.H.H.); a.y.vik@odont.uio.no (A.Y.); 4 Department of Rheumatology, Oslo University Hospital, 0317 Oslo, Norway; oypalm@gmail.com; 5 Department of Hematology, Oslo University Hospital, 0317 Oslo, Norway; 6 Division of Human Nutrition, Faculty of Medicine and Health Sciences, Stellenbosch University, 7505 Tygerberg, South Africa

**Keywords:** body composition, dietary intakes, oral health, primary Sjögren’s syndrome

## Abstract

There is limited knowledge about dietary intake and body composition among patients with primary Sjögren’s syndrome. We assessed dietary intakes with 24-h recalls and body composition with anthropometry and bioelectrical impedance in 20 female patients. Various scoring tools were used to assess oral health. The patients had a lower energy percentage (E%) from carbohydrates (*p* = 0.02) and a higher E% from fat (*p* = 0.01) compared to a reference group. The lower intake of carbohydrates was due to a lower bread intake (*p* = 0.04), while the higher intake of fat was due to a higher intake of butter, margarine, and oil (*p* = 0.01). The patients ate more than twice (*p* = 0.02) as much fish as the reference group. The compliance to recommended intakes of macro- and micronutrients was good. Forty-percent of the patients were overweight/obese. Increased intake of beverages was observed in patients with severe xerostomia and/or low oral health-related quality of life, whereas reduced fat intake was found in hyposmic patients. In conclusion, the dietary intake among the patients was not much different from the reference group and complied with recommendations. Most oral health parameters were not associated with nutrient intakes. Specific dietary guidelines are probably not needed to ensure adequate nutrition among such patients.

## 1. Introduction

Primary Sjögren’s syndrome (pSS) is an autoimmune disorder of the exocrine glands with unknown etiology, and it mainly affects postmenopausal women [1]. The prevalence of pSS depends on age and classification criteria [2], and in Norwegians it is estimated at 0.05% when applying the criteria of the American-European Consensus Group [3].

Patients with pSS display a wide range of symptoms and low secretion of saliva, which, in particular, contributes to increased risk of dental caries and oral infections. Moreover, digestive manifestations with dysphagia and dysmotility of the pharynx are common, and both the pancreas and the liver may be affected [4,5]. There is also a higher risk of gastroesophageal reflux disease among pSS-patients compared with the general population [6].

These manifestations may affect food intake among pSS patients. Rhodus found significantly lower intakes of twelve analysed nutrients compared both with healthy controls and with the Recommended Daily Average values [7]. Contrary to this, Hay et al. did not find that pSS-patients had a diet different from the recommended intakes of the New Zealand population, even though the same patient group reported an altered food intake after the onset of xerostomia [8]. Cermak et al. reported that women with pSS had a significantly higher intake of energy, glutamate, carbohydrates, lactose, phosphorus, caffeine, thiamine, and riboflavin compared with age-matched controls [9]. Moreover, Tovar et al. indicated that patients with pSS were deficient in vitamin B_6_, probably due to a limited intake and not to abnormal uptake mechanisms [10], and Erten et al. found that pSS-patients were deficient in vitamin D [11].

There are only a few dietary intervention trials involving pSS-patients, and these have mostly investigated salivary secretion. For example, Singh et al. found no significant effects of supplementation of omega-3 fatty acids and vitamin E on salivary secretion flow compared with placebo oil [12]. A Norwegian study investigated the effect of a liquid diet in pSS-patients and found that the intervention group had increased salivary flow compared to controls after 4 weeks on the diet [13].

The reduced ability to taste and smell in pSS contributes to decreased appetite and impaired secretion of saliva, which may cause difficulties when eating and swallowing [14]. Rusthen et al. found that many pSS-patients have an impaired taste perception and, in addition, often have difficulty in eating dry foods [15,16]. Moreover, significant differences in body composition between pSS-patients and controls have been reported [17,18,19].

Currently, there is limited data about what pSS-patients actually eat, but such information is necessary for diet-recommendations. We therefore examined the dietary intake among a well-characterized cohort of Norwegian female pSS-patients and compared it with the dietary intake of a national reference group and with the Nordic dietary recommendations. In addition, we studied associations between dietary intake/body composition and oral health among the patients.

## 2. Materials and Methods

### 2.1. Study Patients

In this cross-sectional study, we recruited sequential and unselected pSS-patients from the Dry Mouth Clinic at the Faculty of Dentistry, University of Oslo, Norway. All patients were classified according to the criteria of the American-European Consensus Group [20], and they gave their informed consent for inclusion before they participated in the study. The study, which lasted from January to May 2017, was conducted in accordance with the Declaration of Helsinki, and the protocol was approved by The Regional Committee for Medical and Health Research Ethics (#2015/363).

### 2.2. Measurement of Dietary Intakes

Three repeated 24-h recall interviews (two on week-days, one on a week-end) were performed to assess the dietary intake. The patients were asked, through a structured interview, about what they ate in the past 24 h. These interviews were performed using an in-house data program (KBS version 7) [21]. The first interview was performed during a study visit, whereas the two following interviews were conducted by telephone, with an interval of 4–6 weeks between each interview. The patients were supplied with a booklet with pictures of food portions sizes for guidance during the interviews.

### 2.3. The Reference Group 

The dietary data were compared to an age- and gender-matched reference group from the dietary survey Norkost 3 (N3), which was conducted among Norwegian adults in 2010–2011 [22]. Information on diet in the N3 was collected from 862 men and 925 women, aged 18 to 70 years, by the use of two 24-h recalls, using KBS. The female participants matching the pSS-patients in age—761—were extracted from the N3 population (N3-reference group).

### 2.4. The Nordic Recommendations for Dietary Intakes

We examined whether the pSS-patients complied with the Nordic Nutrition Recommendations for dietary intakes using the guidelines for comparisons as stated in these recommendations [23], which are also recommended by the Norwegian Health Authorities.

### 2.5. Anthropometric Measurements

Triceps skinfold thickness (TSF; ±0.1 mm) was measured with a calliper (Harpenden, UK), and mid upper arm circumference (MUAC; ±1 mm) was measured with a non-stretchable tape at the midpoint between acromion and olecranon of the non-dominant arm. Mid upper arm muscle circumference (MUAMC) was calculated as: MUAMC = MUAC (3.14 × TSF × 0.1), and reflects bone- and muscle mass of the upper arm [24]. Moderate malnourishment was classified as a TSF < 12 mm and/or MUAMC < 19 cm, whereas severe malnourishment corresponded to a TSF < 10 mm and/or MUAMC < 18 cm [24]. Waist circumference was measured with a non-stretchable tape placed horizontally between the iliac crest and the lowest rib. The patients were classified according to WHO-guidelines, in which waist circumference for women is normal (<80 cm), moderately increased (80–88 cm), or greatly increased (>80 cm) [25]. Height (±1 mm) was measured by a stadiometer (SECA GmBH, Hamburg, Germany). BMI was calculated as weight/(height)^2^, and the patients were classified as underweight (<18.5 kg/m^2^), normal weight (18.5–24.9 kg/m^2^), overweight (25–29.9 kg/m^2^), obese class I (30–34.9 kg/m^2^), obese class II (35–39.9 kg/m^2^), or obese class III (>40 kg/m^2^).

### 2.6. Bioelectrical Impedance Analysis

Weight, fat mass, fat-free mass, visceral fat, and total body water were used to describe the body composition and measured with the bioelectrical impedance analyser SECA mBCA 515 (SECA, GmBH).

### 2.7. Oral Measurements

The Oral Health Impact Profile (OHIP)-14 was applied to collect information on oral health-related quality of life (OHRQoL) [26]. The OHIP-14 score has a value between 0 and 56, in which higher scores indicate poorer OHRQoL. The Summated Xerostomia Inventory–Dutch Version (SXI-D) [27] was used to determine subjective feeling of oral dryness; the scores range from 5 to 15, and high scores indicate more severe dryness. The Clinical Oral Dryness Score (CODS) gives an objective score for oral dryness; the scores range from 0 to 10, and higher scores indicate more severe dryness [28]. Secretion of saliva was measured using standardized protocols; unstimulated whole saliva (UWS) was collected for 15 min, and then stimulated whole saliva (SWS) was collected for five minutes while chewing a paraffin wax tablet (Ivoclar Vivadent, Schaan, Lichtenstein). Normal UWS secretion rate is >1.5 mL/15 min [29], and normal SWS secretion rate is >3.5 mL/5 min [30]. Olfactory testing was performed using 12 different odour pens (Sniffin’ Sticks-Screening; Burghart Messtechnic, Wedel, Germany), and patients were classified as anosmic (complete loss of smell, score 0–5), hyposmic (reduced ability to smell, scores 6–9), or normosmic (scores 10–12). Gustatory testing was performed using strips with the taste qualities of sweet, sour, salty, and bitter in four different concentrations (Burghart Messtechnik), and patients were classified as ageusic (complete loss of taste, scores 0–12), hypogeusic (partial loss of taste, scores 13–18), or normogeusic (scores 19–32) [15].

### 2.8. Statistical Analyses

Since we wanted to describe dietary intakes, body composition, and oral health parameters, no sample size calculation was performed. Normality of the data-sets was decided from Shapiro-Wilk’s test, histograms, and normality plots, and presented as means and standard deviation (SD), whereas non-normally distributed data are given as medians and interquartile ranges (IQR). For between-groups comparisons, a t-test or a Mann-Whitney U test was applied, as appropriate. Paired samples t-test or Wilcoxon signed rank test, as appropriate, were applied to investigate differences within a group. SPSS version 24 was used for the statistical analyses. The level of significance was set at 0.05. The study was intended to be explorative, and no corrections of *p*-values were performed for multiple-testing.

## 3. Results

### 3.1. Characteristics of the pSS-Patients

Among the 40 invited pSS-patients, 20 were enrolled. Those who declined gave time constraints and illness as main reasons for not to participate. Sociodemographic characteristics are presented in Table 1.

### 3.2. Dietary Intakes Recorded by 24-h Recalls

The dietary intakes of main food categories are given in Table 2. The pSS-patients had a significantly higher intake of fish, butter, margarine and oil, sugar and sweets, and spices compared with the N3-reference group, whereas the latter had a significantly higher intake of bread.

The daily energy intake was similar (P = 0.42) in the pSS- and the N3-reference groups (Table 3).

Whereas the percent energy intake (E%) from carbohydrates was significantly lower among the pSS-patients compared with the N3-reference group, their E% from total fat, monounsaturated fat, and alcohol was significantly higher. The absolute mean intake (excluding supplements) of vitamins A, E, B_6_, B_12_, C, thiamine, riboflavin, potassium, magnesium, zinc, copper, and phosphate was above the recommended intake values, whereas that of vitamin D, folate, calcium, iron, and selenium were below (Table 4). For both the pSS-patients and the N3-reference group, the micronutrient-intake increased when supplements were included in the analyses. However, for some nutrients such as vitamin D and calcium, the median intakes increased to a level above the recommendations when the pSS-patients took supplements, while for other nutrients such as folate and iron, intake remained below. The absolute intake of vitamin D (excluding supplements) was higher in the pSS- (*p* = 0.02) compared to the N3-reference group. With supplements included, the pSS-patients had a higher intake of vitamin D (*p* < 0.01), vitamin B_6_ (*p* = 0.04), and magnesium (*p* < 0.01) compared to the N3-reference group.

The pSS-patients had a higher median intake adjusted per 10 MJ of vitamin D (*p* = 0.02) and vitamin E (*p* = 0.03), excluding supplements (Supplementary Table S1), while the N3-reference group had a higher intake of thiamine (*p* = 0.01) and riboflavin (*p* = 0.01).

The food sources of energy—macro- and micronutrients—among the pSS-patients are presented in Supplementary Tables S2–S4. Bread, fruits, berries, and meat were the main sources of energy, whereas the main sources of protein were meat, fish, and cheese. Butter, margarine and oil, cheese, and meat were the main sources of fat, while bread, fruits, berries, and grains were the main sources of carbohydrate. Bread, grains, and potatoes contributed to the intake of starch, while bread, fruits, berries, and vegetables were the main sources of dietary fibre. The main sources of sucrose were sugar and sweets, milk products, and cakes. All of the studied vitamins, except beta-carotene, had supplements as the largest source of intake.

### 3.3. Patients’ Compliance with the Nordic Recommendations for Nutritional Intakes

The pSS-patients showed the highest compliance with the recommended intakes for red meat and the lowest for intakes of vegetables (Supplementary Table S5). Approximately half of the patients complied with the recommendation for intake of fruits, berries, and fish, whereas about 1/3 complied with the intake-recommendation for fatty fish.

There was good compliance with the recommendations for nutrient intakes (Supplementary Table S6). The compliance with the recommendations for vitamin D, folate, and iron were low. For most of the other micronutrients, there was a high proportion of compliance with the recommendations. Notably, for trans-unsaturated fat and monounsaturated fat, all patients complied with the recommendations.

### 3.4. Body Composition of the Patients

According to BMI, MUAMC, or TSF, none of the pSS-patients was classified as underweight or as having malnutrition (Table 5). Twelve of the patients were classified as having a normal body weight, five were overweight, two were obese class I, none were obese class II, and one was obese class III. Seven patients had a normal waist circumference, four had a moderately increased waist circumference, and nine had a greatly increased waist circumference.

### 3.5. Oral Health Parameters

Fifteen patients had an OHIP-14 score ≥8; 16 of them reported severe xerostomia (SXI-D ≥ 10); and 12 had CODS scores ≥5, indicating severe oral dryness. Seventeen had pathologically low UWS secretion rate, while ten had pathologically low SWS secretion rate (Table 6). Olfactory scores classified one as anosmic, nine as hyposmic, and ten as normosmic. Gustatory scores classified six as ageusic, four as hypogeusic, and ten as normogeusic.

When comparing patients with the lowest to the highest UWS secretion rate and those with the lowest to the highest SWS secretion rate regarding intake of the various food categories or nutrients, no significant differences were found.

The group with OHIP-14 scores ≥8 had a higher intake of beverages (*p* = 0.02) than those with lower scores. Similarly, there was a higher intake of beverages in the group with SXI-D scores ≥10 (*p* = 0.03). The group with CODS scores ≥5 had a higher intake of fruits and berries (*p* = 0.01) and a higher intake of grains (*p* = 0.04) than the group with lower scores. Regarding smell, the group classified as hyposmic had a lower intake of fruits and berries (*p* = 0.03) and of vegetables (*p* = 0.05) than the normosmic. Moreover, we found a lower intake of energy (*p* = 0.03) and total fat (*p* = 0.04) among the hyposmic. The intake of various food catogories did not (*p* > 0.05) influence tasting, but the ageusic had a lower intake (*p* = 0.02) of supplemental vitamin D than the normogeusic.

## 4. Discussion

The pSS-patients had a higher absolute intake of fat and a lower intake of carbohydrates compared to the N3-reference group. In addition, the mean E% from carbohydrates among pSS-patients was below the recommendations. A higher intake of butter, margarine, and oil probably contributed to the higher intake of fat, while a lower intake of bread contributed to the lower intake of carbohydrates. Notably, the daily intake of fish among the pSS-patients was more than twice that of the N3-reference group. None of the pSS-patients was underweight or malnourished, whereas 40% were overweight/obese.

A possible explanation for the higher intake of fat-products among the pSS-patients may be a lubricating effect in the oral cavity, thereby aiding mastication and swallowing [31]. Impaired taste function can be another explanation, as patients experiencing taste changes have reported change in eating habits to compensate for the missing sensory experience [32].

The high intake of lozenges among the pSS-patients was expected, as they stimulate salivary secretion, whereas the high intake of chocolate might be due to a higher demand for sweet taste caused by disturbed gustatory function. Rusthen et al. showed that 19% of the pSS patients were aguesic, 32% were hypogeusic, 13% were anosmic, and 29% were hyposmic, which could affect taste [15]. A Mexican study concluded that pSS-patients were only mildly dysgeusic to the taste of sweet and salt, and more dysgeusic to sour and bitter tastes [33]. The reduced perception of smell and flavour among the pSS-patients may have led to an increased need to add spices to the food [34]. Five of our patients avoided spicy food, but there were no significant differences in the intake of spices between ageusic/hyposmic versus normosmic patients.

Although milk may function as a saliva substitute [35], Cermak et al. found a significantly higher intake of glutamate, lactose, thiamine, and riboflavin among pSS-patients, which was ascribed to a higher intake of milk [9]. However, this finding was not supported by the current study. The fine, smooth, and viscous texture of fish foods compared with meat may facilitate eating and swallowing among the pSS-patients, thus explaining their higher fish intake compared with the N3-reference group.

Compliance with the nutrient-recommendations, excluding supplements, was good overall, with exceptions for saturated fat, fibre, vitamin D, folate, and iron, which is in agreement with the general Norwegian female population [22]. The results also indicate that the pSS-patients have a sufficient intake of micronutrients.

The pSS-patients had a lower E% from carbohydrate compared with the N3-reference group and with the recommendations. In contrast, Cermak et al. found higher carbohydrate intake in pSS-patients compared with controls [9], and Hay et al. found that carbohydrate intake in pSS-patients complied with recommendations [8]. The low intake of starch could be expected, as many starch foods are considered as dry. The intake of E% from total fat among our patients was within the recommended intake limits, while the absolute fat intake and the intake of E% from monounsaturated fat were higher than in the N3-reference group. The mean E% from saturated fat above the recommended limit of 10 E% among the pSS-patients is similar to that of the Norwegian population [36]. The median intake (about 3 g/day) of omega-3 fatty acids was higher in the pSS-patients (with and without supplements) compared with the N3-reference group, possibly in part explained by the higher fish intake. Reportedly, an intake above 3–4 g/day reduces the need for anti-inflammatory drugs in rheumatic patients and may be an explanation for the higher intake observed in our pSS-patients [37].

The intake of vitamin D among the pSS-patients were higher than in the N3-reference group. However, the pSS-patients had a median dietary intake of 5.4 µg/day, and when including supplements it increased to 12.7 µg/day, indicating that there is a need for vitamin D supplementation. Whereas Cermak et al. did not find a different intake of vitamin D between pSS-patients and controls [9], Erten et al. found lower plasma levels of vitamin D in patients compared with controls [11]. A recent review [38] found evidence of lower vitamin D levels in pSS-patients that were associated with extraglandular manifestations, whereas another study did not [39].

We found that 40% of the pSS-patients were overweight/obese (BMI > 25 kg/m^2^). The mean BMI was 25.4 kg/m^2^, whereas the mean BMI of all the women in the N3 (not just the N3-reference group) was 24.6 kg/m^2^. The limited sample size prevented us from examining statistical associations between the overweight/obese and their intakes, but they appeared to have lower intake of micronutrients and higher energy intake from fat than normal weight patients. The patients studied by Rhodus had a mean BMI of 23.2 kg/m^2^, which was significantly lower than the BMI of 25 kg/m^2^ among the controls [7], while a New Zealand pSS-population had a mean BMI of 25.5 kg/m^2^ [8]. The ethnicities of these populations may be different from ours, thus precluding direct comparisons.

Dysgeusia did not influence the intake of the food categories or nutrients, and neither did the salivary flow. There was a higher intake of beverages in the group with more severe xerostomia as indicated by SXI-D, and in the group with lowest OHRQoL. The hyposmic patients had a lower intake of fat, which is in accordance with Kong et al., in which there was a lower intake of fat among women with olfactory dysfunction [40]. The opposite was found by Duffy et al., in which the women with the best olfactory function had a lower E% intake from total fat [41].

The main limitations of our study are the small sample size, the lack of biomarkers for nutrient intakes, and no control group regarding the oral parameters. Major strengths include the inclusion of a matched reference group and the detailed information of dietary intakes, body composition, and a wide range of oral health parameters.

The dietary intakes of foods and nutrients among the pSS-patients were not much different from the reference group, and the overall compliance with the Nordic dietary recommendations was fairly good. Salivary secretion was not associated with specific food or nutrient intakes, but consumption of beverages was highest among those with severe oral dryness. Specific dietary guidelines are probably not needed to ensure adequate nutritional health among pSS-patients.

## Figures and Tables

**Table 1:** Sociodemographic characteristics of the 20 pSS-patients.

Characteristic	Value
Mean (range) age (years)	54.1 (34–70)
Mean (range) number of drugs	2.1 (0–6)
Education	
Primary and lower secondary school	1 (5)
Upper secondary school	6 (30)
College/university	13 (65)
Work	
Full time	8 (40)
Part time	5 (25)
Not working	7 (35)
Marital status	
Married/cohabitant	13 (65)
Single	7 (35)
Smoker	
Yes	4 (20)
No	16 (80)

Values are numbers (%) unless otherwise stated.

**Table 2:** Intake of food categories among the pSS-patients and the N3 reference group.

Food Category (g/day)	pSS (*n* = 20)	N3 (*n* = 761)	*p*-Value
Bread	93 (83)	123 (91)	0.04
Grains	67 (91)	45 (109)	0.25
Cakes	17 (31)	15 (54)	0.92
Potatoes	56 (65)	49 (90)	0.71
Vegetables	142 (153)	143 (122)	0.69
Fresh fruits and berries	127 (157)	150 (195)	0.58
Jam	9 (21)	5 (20)	0.24
Juice ^#^	78 (123)	101 (151)	0.77
Nuts, olives and seeds ^#^	6 (10)	5 (12)	0.10
Meat	69 (98)	90 (95)	0.31
Fish	77 (114)	29 (91)	0.02
Egg	19 (53)	5 (38)	0.72
Milk products	138 (255)	165 (284)	0.88
Cheese	26 (34)	32 (40)	0.79
Butter, margarine, oil	23 (16)	15 (22)	0.01
Sugar and sweets	19 (16)	9 (22)	0.01
Beverages	1773 (1020)	2066 (1178)	0.34
Spices	1.0 (1.3)	0.0 (0.5)	0.01
Various items	22 (35)	15 (45)	0.27
Sauces	19 (36)	10 (33)	0.21

Values are medians (interquartile range). pSS: primary Sjögren’s syndrome. ^#^ Values presented as mean and standard deviation (SD).

**Table 3:** Intake of energy and macronutrients among the pSS-patients and the N3 reference group.

	pSS (*n* = 20)		N3 (*n* = 761)		Recommended ^a^	
	g/day	E%	g/day	E%	E%	
	Mean ± SD	Mean ± SD	Mean ± SD	Mean ± SD		*p-*Value ^b^
Energy, kJ/d	8245 ± 2091		7802 ± 2436			0.42
Carbohydrate	184 ± 53.3	38.8 ± 9.1	200 ± 72.1	43.3 ± 8.0	45–60	0.02
Sugar	28.8 ± 12.6	5.4 (3.5)	27.1 (30.4)	6.0 (5.9)	<10	0.89
Fat	87.0 ± 31.0	38.4 ± 5.8	72.4 ± 29.2	34.4 ± 7.1	25–40	0.01
Saturated fat	31.5 (19.3)	14.4 ± 4.0	25.8 (15.4)	13.1 ± 3.5	<10	0.09
Trans-unsaturated fat	0.7 (0.6)	0.4 (0.2)	0.7 (0.5)	0.3 (0.3)	<1	0.81
Monounsaturated fat	32.0 ± 10.2	14.2 ± 2.2	26.3 ± 11.6	12.5 ± 3.4	10–20	0.02
Polyunsaturated fat	13.9 ± 4.7	6.3 ± 1.4	11.6 ± 5.5	5.7 ±1.9	5–10	0.17
Omega 3	3.0 (2.8)		2.0 (1.5)		1	<0.01
Omega 6	10.7 ± 3.5		9.4 ± 4.4			0.17
Alcohol	7.1 (18.6)	2.8 (6.2)	0.0 (8.2)	0.0 (3.2)	<5	<0.01
Fibre	20.9 ± 6.0		22.0 ± 8.2		25 g/day	0.52

Values are medians (interquartile range) or means ± SD. Supplements are excluded. ^a^ Nordic Nutrition Recommendations. ^b^ *p*-values for differences between the pSS- and the N3-reference group. pSS: primary Sjögren’s syndrome; E%: percent of energy intake.

**Table 4:** Intake of micronutrients among the pSS-patients and the N3-reference group.

Nutrient, Unit	pSS (*n* = 20)		N3 (*n* = 761)		Recommended ^a^
	Median (IQR)		Median (IQR)		
	No Supplements	With Supplements	No Supplements	With Supplements	
Vitamin A, µg/day	796 (423)	991 (767)	690 (398)	820 (578)	700
Retinol, mg/day	356 (339)	549 (452)	381 (318)	472 (463)	
Beta-carotene, mg/day	2764 (2283)	2768 (2009)	2282 (2672)	2342 (2700)	
Vitamin D, µg/day	5.4 (5.8)	12.7 (15.2)	4.4 (4.1)	6.5 (8.9)	10
Vitamin E, mg/day	13.6 (4.9)	17.1 (9.1)	11.3 (6.1)	14.7 (12.4)	8
Thiamine, mg/day	1.3 (0.6)	1.8 (1.1)	1.4 (0.7)	1.6 (0.9)	1.1 (1.0)
Riboflavin, mg/day	1.7 (0.8)	2.1 (1.0)	1.8 (0.9)	2.1 (1.2)	1.2
Niacin, mg/day	17.8 (9.2)	23.7 (12.9)	17.5 (9.6)	20.2 (12.4)	14 (13)
Vitamin B_6_, mg/day	1.6 (0.7)	2.0 (2.3)	1.4 (0.7)	1.7 (1.1)	1.2 (1.3)
Folate, µg/day	256 (111)	289 (143)	241 (110)	264 (134)	400 (300)
Vitamin B_12_, µg/day	4.9 (5.2)	6.0 (5.6)	5.1 (3.7)	5.5 (4.3)	2
Vitamin C, mg/day	77 (70)	128 (134)	97 (89)	113 (105)	75
Calcium, mg/day	751 (548)	872 (546)	775 (518)	795 (511)	800
Iron, mg/day	9.3 (4.1)	9.4 (6.4)	9.0 (4.3)	9.5 (5.2)	15 (9)
Sodium, g/day	2.6 (1.1)	2.6 (1.1)	2.3 (1.2)	2.3 (1.2)	
Potassium, g/day	3.3 (1.3)	3.3 (1.3)	3.5 (1.3)	3.5 (1.3)	3.1
Salt, g/day	6.5 (2.6)	6.5 (2.6)	5.7 (3.0)	5.7 (3.0)	
Magnesium, mg/day	333 (79)	387 (236)	316 (149)	336 (155)	280
Zinc, mg/day	9.1 (4.6)	10.7 (6.2)	9.7 (4.5)	10.5 (5.5)	7
Copper, mg/day	1.1 (0.4)	1.2 (0.7)	1.0 (0.5)	1.1 (0.6)	0.9
Phosphate, mg/day	1434 (457)	1434 (457)	1451 (630)	1462 (626)	600
Selenium, mg/day	45.0 (37.0)	51.0 (51.0)	42.0 (27.0)	48.0 (31.0)	50

^a^ According to the Nordic Nutrition Recommendations, for women aged 31–60 years or (61–74 years). pSS: primary Sjögren’s syndrome; IQR: interquartile range.

**Table 5:** Anthropometric and body composition measurements of the pSS-patients.

Characteristic	Median (IQR)
Height, m	1.67 (0.1)
Weight, kg	69.4 (13.4)
Body mass index, kg/m^2^	23.3 (4.3)
Waist circumference, m	0.85 (0.19)
Mid upper arm muscle circumference, cm	28.3 (5.8)
Triceps skinfold thickness, mm	20.1 (5.8)
Arm muscle circumference, cm	22.1 (3.5)
Fat mass, %	36.5 (14.5)
Fat mass index, kg/m^2^	8.5 (4.7)
Fat-free mass, %	63.6 (14.5)
Fat-free mass index, kg/m^2^	15.7 (1.3)
Visceral adipose tissue, litres	1.3 (1.3)
Total body water, %	47.2 (10.0)

pSS: primary Sjögren’s syndrome, IQR: interquartile range.

**Table 6:** Oral health parameters among the pSS-patients.

Variable	Median (IQR)
Unstimulated whole saliva, mL per 15 min	0.78 (1.18)
Stimulated whole saliva, mL per 5 min	3.49 (3.32)
Olfactory score	9.5 (2.8)
Gustatory score	19.0 (13.5)
Clinical Oral Dryness Score	5.0 (2.8)
Oral Health Impact Profile sum score	14.0 (20.3)
Summated Shortened Xerostomia Inventory	12.0 (4.8)
pSS: primary Sjögren’s syndrome, IQR: interquartile range.	

## Supplementary Materials

The following are available online at http://www.mdpi.com/2072-6643/10/7/866/s1, Table S1: intake of micronutrients per 10 MJ among the pSS-patients and the N3-reference group; Table S2: sources of energy and macronutrients for the pSS-patients; Table S3: sources of vitamins for the pSS-patients; Table S4: sources of minerals for the pSS-patients; Table S5: compliance of the pSS-patients with the Nordic recommendations for food intakes; and Table S6: proportions of the pSS-patients and the N3-reference group complying with the Nordic recommendations for nutrient intakes.
