# Supplementary material for: Dietary Intake, Body Composition, and Oral Health Parameters among Female Patients with Primary Sjögren’s Syndrome
Source: Nutrients. 2018 Jul 4;10(7):866. doi: 10.3390/nu10070866 (PMC6073564; doi:10.3390/nu10070866)
Supplement: Supplementary file 1 [file nutrients-10-00866-s001.zip › Table S1.pdf]

Supplementary Table 1 Intake of micronutrients per 10 MJ among the pSS-patients and the N3-reference group

	**pSS (n=20) Median (IQR)**		**N3 (n=761) Median (IQR)**	
**Nutrient, unit**	**No supplements**	**With supplements**	**No supplements**	**With supplements**
Vitamin A, µg/d	903 (446)	1259 (857)	899 (447)	1067 (680)
Retinol, mg/d	524 (336)	639 (706)	499 (331)	622 (523)
Beta-carotene, mg/d	3063 (2662)	3185 (2400)	2946 (3814)	3040 (3825)
Vitamin D, µg/d	6.6 (5.3)	14.7 (13.2)	5.9 (4.9)	8.8 (10.7)
Vitamin E, mg/d	16.1 (3.3)	23.1 (12.3)	14.4 (6.0)	19.1 (14.3)
Thiamine, mg/d	1.6 (0.4)	2.2 (1.8)	1.8 (0.6)	2.0 (1.0)
Riboflavin, mg/d	1.9 (0.7)	2.6 (1.9)	2.3 (0.9)	2.6 (1.3)
Niacin, mg/d	22.2 (10.1)	28.2 (28.2)	22.9 (10.2)	25.8 (14.0)
Vitamin B_6, mg/d	1.9 (1.0)	2.4 (2.7)	1.8 (0.8)	2.1 (1.4)
Folate, µg/d	314 (129)	351 (148)	305 (100)	338 (146)
Vitamin B_{12}, µg/d	5.7 (6.5)	7.1 (8.2)	6.9 (4.3)	7.5 (4.9)
Vitamin C, mg/d	99 (61)	151 (204)	130 (117)	154 (137)
Calcium, mg/d	997 (320)	1012 (593)	1011 (532)	1053 (557)
Iron, mg/d	10.9 (4.1)	11.5 (5.4)	11.6 (3.7)	12.2 (5.0)
Sodium, g/d	3.1 (1.1)	3.1 (1.2)	3.0 (1.3)	3.0 (1.3)
Potassium, g/d	4.3 (1.1)	4.2 (1.1)	4.5 (1.5)	4.6 (1.5)
Salt, g/d	8.0 (2.7)	8.0 (2.7)	7.4 (3.0)	7.4 (3.0)
Magnesium, mg/d	386 (131)	524 (340)	415 (119)	429 (132)
Zinc, mg/d	12.0 (3.3)	12.5 (4.9)	12.6 (4.0)	13.4 (5.4)
Copper, mg/d	1.5 (0.3)	1.5 (0.7)	1.4 (0.5)	1.4 (0.6)
Phosphate, mg/d	1831 (418)	1828 (401)	1897 (528)	1900 (539)
Selenium, mg/d	60.5 (51.0)	63.5 (65.0)	54.0 (30.0)	59.2 (40.0)

pSS primary Sjögren's syndrome, *IQR* interquartile range.
