# Supplementary material for: Dietary Intake, Body Composition, and Oral Health Parameters among Female Patients with Primary Sjögren’s Syndrome
Source: Nutrients. 2018 Jul 4;10(7):866. doi: 10.3390/nu10070866 (PMC6073564; doi:10.3390/nu10070866)
Supplement: Supplementary file 1 [file nutrients-10-00866-s001.zip › Table S2.pdf]

Supplementary Table 2 Sources of energy and macronutrients for the pSS-patients

Food group	Energy	Protein	Fat	Saturated fat	Unsaturated fat			Cholesterol	Omega 3	Omega 6	Carbo-hydrates	Starch	Fibre	Sugar
					Trans-	Mono-	Poly-							
Bread	14	12	5	2	0	4	10	0	7	11	27	53	28	2
Grains	7	6	4	1	0	4	8	0	6	8	13	17	13	5
Cakes	4	2	5	4	3	6	4	5	3	5	6	7	3	13
Potatoes	3	1	2	1	3	2	2	0	1	2	6	11	5	0
Vegetables	4	3	3	2	2	4	4	0	3	5	4	1	18	2
Fruits and berries	10	4	5	2	1	6	11	0	5	11	18	3	20	12
Meat	9	22	13	13	8	15	10	17	5	11	1	1	0	0
Fish	8	18	9	6	2	10	16	16	36	9	3	3	2	0
Egg	2	4	4	3	1	4	3	37	2	4	0	0	0	0
Milk products	7	8	7	11	15	5	1	4	1	1	8	1	1	19
Cheese	8	13	14	25	46	9	2	11	2	2	1	0	0	0
Butter, margarine, oil	7	1	18	16	15	21	20	6	17	22	0	0	0	1
Sugar and sweets	4	1	5	7	2	4	1	1	0	2	6	0	4	29
Beverages	8	3	1	2	0	1	0	1	0	0	6	1	1	11
Supplements	1	0	1	1	0	2	3	1	10	0	0	0	0	0
Spices	0	0	0	0	0	0	0	0	0	0	0	0	1	0
Various items	3	1	4	3	1	5	4	0	1	5	3	2	2	4

Values are percentages of total intake per day. *pSS* primary Sjögren's syndrome.
