# Supplementary material for: Dietary Intake, Body Composition, and Oral Health Parameters among Female Patients with Primary Sjögren’s Syndrome
Source: Nutrients. 2018 Jul 4;10(7):866. doi: 10.3390/nu10070866 (PMC6073564; doi:10.3390/nu10070866)
Supplement: Supplementary file 1 [file nutrients-10-00866-s001.zip › Table S3.pdf]

Supplementary Table 3 Sources of vitamins for the pSS-patients

**Food group**	Vitamin A	Retinol	Beta-carotene	Vitamin D	Vitamin E	Thiamine	Riboflavin	Niacin	Vitamin B_6	Folate	Vitamin B_{12}	Vitamin C
Bread	0	1	0	0	6	13	4	8	5	11	0	0
Grains	0	0	0	0	3	5	2	3	3	3	0	0
Cakes	2	4	0	2	4	2	1	1	1	2	0	0
Potatoes	1	1	2	0	2	2	1	4	3	3	0	4
Vegetables	19	2	81	1	7	4	2	5	5	13	0	26
Fruits and berries	1	0	3	0	6	7	6	6	13	12	0	31
Meat	8	13	0	2	3	13	7	21	10	2	4	0
Fish	3	3	1	18	8	4	4	13	10	3	7	0
Egg	6	10	0	6	8	2	6	0	1	5	2	0
Milk products	5	8	1	1	1	4	10	1	2	4	2	1
Cheese	13	7	1	0	1	1	6	1	1	4	2	0
Butter, margarine, oil	14	23	3	9	13	0	1	0	0	0	0	0
Sugar and sweets	0	0	0	0	1	1	2	0	0	0	0	0
Beverages	1	1	0	0	0	2	11	9	3	2	1	1
Supplements	25	24	4	61	34	39	36	26	40	33	81	34
Spices	0	0	0	0	0	0	0	0	0	0	0	0
Various items	1	1	2	0	4	1	1	2	2	2	0	1

Values are percentages of total intake per day. *pSS* primary Sjögren’s syndrome.
