# Supplementary material for: Dietary Intake, Body Composition, and Oral Health Parameters among Female Patients with Primary Sjögren’s Syndrome
Source: Nutrients. 2018 Jul 4;10(7):866. doi: 10.3390/nu10070866 (PMC6073564; doi:10.3390/nu10070866)
Supplement: Supplementary file 1 [file nutrients-10-00866-s001.zip › Table S4.pdf]

Supplementary Table 4 Sources of minerals for the pSS-patients

**Food group**	Calcium	Iron	Sodium	Potassium	Salt	Magnesium	Zinc	Selenium	Copper	Phosphate
Bread	3	16	16	7	15	12	12	7	14	12
Grains	3	8	5	4	6	7	5	2	9	7
Cakes	1	3	2	1	2	2	2	2	3	3
Potatoes	1	3	1	8	1	3	1	0	4	2
Vegetables	4	7	3	15	3	5	4	0	9	5
Fruits and berries	5	8	2	18	1	11	4	2	15	5
Meat	2	12	17	9	16	4	20	12	5	12
Fish	4	4	13	10	13	5	5	43	6	11
Egg	2	6	2	1	2	1	3	8	1	5
Milk products	23	2	3	8	3	5	6	3	3	12
Cheese	32	1	9	2	9	3	13	6	2	15
Butter, margarine, oil	0	0	5	0	5	0	0	1	0	1
Sugar and sweets	2	3	0	1	0	2	1	0	4	2
Beverages	7	8	4	13	4	9	2	1	4	6
Supplements	7	16	0	0	0	29	19	13	19	0
Spices	0	1	5	0	5	0	0	0	0	0
Various items	2	3	14	3	14	2	1	1	3	2

Values are percentages of total intake per day. *pSS* primary Sjögren's syndrome.
