# Supplementary material for: Dietary Intake, Body Composition, and Oral Health Parameters among Female Patients with Primary Sjögren’s Syndrome
Source: Nutrients. 2018 Jul 4;10(7):866. doi: 10.3390/nu10070866 (PMC6073564; doi:10.3390/nu10070866)
Supplement: Supplementary file 1 [file nutrients-10-00866-s001.zip › Table S5.pdf]

Supplementary Table 5 Compliance of the pSS-patients with the Nordic recommendations for food intakes

**Foods**	**Recommended intake**	**Median (IQR)**	**Compliance %**
Vegetables, fruit and berries	≥500 g/d	365 (299)	30
Vegetables	≥250 g/d	85 (141)	0
Fruits and berries	≥250 g/d	258 (236)	55
Fish, pure[a]	≥300-450 g/week	53 (103)	50
Fatty fish[b]	≥200 g/week	11 (61)	30
Red meat, pure + processed[c]	<500 g/week	46 (69)	70
