# Supplementary material for: Dietary Intake, Body Composition, and Oral Health Parameters among Female Patients with Primary Sjögren’s Syndrome
Source: Nutrients. 2018 Jul 4;10(7):866. doi: 10.3390/nu10070866 (PMC6073564; doi:10.3390/nu10070866)
Supplement: Supplementary file 1 [file nutrients-10-00866-s001.zip › Table S6.pdf]

Supplementary Table 6 Proportions of the pSS-patients and the N3-reference group complying with the Nordic recommendations for nutrient intakes[a]

	**Recommended**	**pSS (*n*=20)** %	**N3 (*n*=761)** %
**Macronutrients, E%**			
Carbohydrate	45-60	30	43
Sugar	<10	85	79
Fat	25-40	65	3
Saturated fat	<10	15	18
Trans-unsaturated fat	<1	100	100
Monounsaturated fat	10-20	100	74
Polyunsaturated fat	5-10	85	58
Protein	10-20	90	78
Fibre, g/d	25	20	33
Alcohol	<5	70	80
**Vitamins, unit**			
Vitamin A, µg/d	700	60	48
Vitamin D, µg/d	10	25	9
Vitamin E mg/d	8	90	77
Thiamine, mg/d	1.1 (1.0)[b]	75	75
Riboflavin, mg/d	1.2	75	85
Niacin, mg/d	14 (13)[b]	80	72
Vitamin B_6, mg/d	1.2 (1.3)	75	68
Folate, µg/d	400 (300)[b]	5	5
Vitamin B_{12}, µg/d	2	95	96
Vitamin C, mg/d	75	55	63
**Minerals, unit**			
Calcium, mg/d	800	45	47
Iron, mg/d	15 (9)[b]	15	8
Potassium, g/d	3.1	70	67
Magnesium, mg/d	280	80	64
Zinc, mg/d	7	85	81
Copper, mg/d	0.9	90	65
Phosphate, mg/d	600	95	99
Selenium, µg/d	50	45	37

[a]Supplements are excluded.

[b]According to the Nordic Nutrition Recommendations for women aged 61-74 years.

pSS primary Sjögren's syndrome, *E%* percent of energy intake.
